# Monitoring anti-PD-1-based immunotherapy in non-small cell lung cancer with FDG PET: introduction of iPERCIST

**DOI:** 10.1186/s13550-019-0473-1

**Published:** 2019-01-29

**Authors:** Lucas Goldfarb, Boris Duchemann, Kader Chouahnia, Laurent Zelek, Michael Soussan

**Affiliations:** 1 Department of Nuclear Medicine, Paris 13 University, APHP, Hôpital Avicenne, 125 rue de Stalingrad, 93000 Bobigny, France; 2 Department of Oncology, Paris 13 University, APHP, Hôpital Avicenne, Bobigny, France; 3 IMIV, CEA, Inserm, CNRS, Université Paris-Sud, Université Paris-Saclay, Orsay, France

**Keywords:** PET, Fluorodeoxyglucose, Non-small cell lung cancer, Nivolumab, iPERCIST

## Abstract

**Background:**

Immunotherapy represents a new therapeutic approach in non-small cell lung carcinoma (NSCLC) with the potential for prolonged benefits. Because of the systemic nature and heterogeneity of tumoral diseases, as well as the immune restoration process induced by immunotherapy, the assessment of therapeutic efficacy is challenging, and the role of FDG PET is not well established. We evaluated the potential of FDG PET to monitor NSCLC patients treated with a checkpoint inhibitor.

**Results:**

This was a retrospective analysis of 28 NSCLC patients treated with nivolumab, a programmed cell death 1 (PD-1) blocker. All patients underwent a PET scan before treatment (SCAN-1) and another scan 2 months later (SCAN-2). Disease progression was assessed by immune PET Response Criteria in Solid Tumors (iPERCIST), which was adapted from PERCIST; and the immune Response Evaluation Criteria in Solid Tumors (iRECIST). iPERCIST is a dual-time-point evaluation of “unconfirmed progressive metabolic disease” (UPMD) status at SCAN-2. UPMD at SCAN-2 was re-evaluated after 4 weeks with SCAN-3 to confirm PMD. Patients with complete/partial metabolic response (CMR or PMR) or stable metabolic disease (SMD) at SCAN-2 or -3 were considered responders. Patients with UPMD confirmed at SCAN-3 were considered non-responders. The Kaplan-Meier method was used to estimate survival. At SCAN-2, we found 9/28 cases of PMR, 4/28 cases of SMD, 2/28 cases of CMR, and 13/28 cases of UPMD. Four of the 13 UPMD patients were classified as responders at SCAN-3 (PMR *n* = 1, SMD *n* = 3). The remaining nine UPMD patients were classified as non-responders due to clinical degradation, and treatment was stopped. The median follow-up was 16.7 months [3.6–32.2]. Responders continued treatment for a mean of 10.7 months [3.8–26.3]. Overall survival was longer for responders than that for non-responders (19.9 vs. 3.6 months, log rank *p* = 0.0003). The 1-year survival rates were 94% for responders and 11% for non-responders. A comparison with iRECIST showed reclassification in 39% (11/28) of patients with relevant additional prognostic information.

**Conclusions:**

iPERCIST dual-time-point evaluation might be a powerful tool for evaluating anti-PD-1-based immunotherapy, with the ability to identify patients who can benefit most from treatment. The prognostic value of iPERCIST criteria should be confirmed in large prospective multicentric studies.

**Electronic supplementary material:**

The online version of this article (10.1186/s13550-019-0473-1) contains supplementary material, which is available to authorized users.

## Background

Lung cancer is one of the most common malignant tumors and represents the leading cause of death by cancer in Europe and in the USA [1, 2]. Non-small cell lung carcinoma (NSCLC) accounts for 85% of lung cancers [3], and close to 70% of patients present with locally advanced or metastatic disease at the time of diagnosis. Recently, immunotherapy has become a standard treatment after chemotherapy in NSCLC, whether locally advanced or metastatic, offering the potential for prolonged response and survival time [4]. Two phase III studies have demonstrated higher overall response and survival rates in patients with NSCLC treated with nivolumab versus docetaxel [5, 6].

Positron emission tomography/computed tomography (PET) with fluorodeoxyglucose (FDG) is widely used to assess the response to chemotherapy. However, the mechanisms of action of checkpoint inhibitors differ from those of chemotherapies. In some cases, the initial response to nivolumab may be an increase in the size and metabolism of tumor lesions due to lymphocyte infiltration, leading to false interpretations [7]. Several response patterns have been described with computed tomography (CT), but data with FDG PET monitoring are scarce. Because of the new mechanisms of action of therapies and the heterogeneity of malignant disease, methods of assessing response are complex, and the role of PET is not well established.

Recently, several attempts to establish adapted imaging criteria as follow-up to immunotherapy treatment have been proposed, notably the immune Response Evaluation Criteria in Solid Tumors (iRECIST) [8]. Regarding metabolic imaging, PET Response Criteria in Solid Tumors (PERCIST) have been proposed for solid tumor monitoring but have not been validated with new immunotherapies. Consequently, the authors proposed other response criteria using a single-time-point PET analysis for immunotherapy response assessment, especially in melanoma patients. Cho et al. studied patients with advanced melanoma under immunotherapy using an early PET evaluation method combined with a CT scan (3–4 weeks) to predict the best overall response at 4 months [9]. The conclusion of the work was that the combination of functional and anatomical imaging parameters obtained early after the start of treatment appears predictive for eventual response at 4 months. Anwar et al. proposed using an absolute number of 4 newly emerged FDG-avid lesions on post-therapy PET scans in melanoma patients under ipilimumab to provide reliable information for treatment response [10, 11]. However, as well-written in the paper from Pinker et al., a new response category, including an indeterminate response, based on a dual-time-point evaluation could be relevant during the metabolic evaluation of immunotherapy, as already shown with iRECIST [12]. This indeterminate response or “unconfirmed” status recognizes that delayed responses or immune-mediated flares can both occur in the first months of immunotherapy treatment [12]. This additional category provides the clinician flexibility to continue treatment in good general status patients and to perform a subsequent evaluation to confirm or deny a truly progressive disease [12].

Based on this assumption, we chose to introduce and evaluate metabolic criteria based on a dual-time-point evaluation, as proposed with iRECIST. We designed a retrospective study to evaluate the prognostic value of FDG PET using modified PERCIST criteria adapted from iRECIST, which we called Immune PET Response Criteria in Solid Tumors (iPERCIST), in monitoring patients with NSCLC treated with an anti-programmed death-1 checkpoint inhibitor.

## Methods

### Patients

Between April 2015 and January 2017, all consecutive patients referred for PET imaging with a diagnosis of NSCLC and who were treated with anti-programmed death-1 checkpoint inhibitor, nivolumab, were retrospectively selected from our database. Patients needed to have undergone a PET scan before the start of the treatment (SCAN-1) and to have at least a second evaluation PET scan 2 months after treatment (SCAN-2).

Nivolumab was prescribed according to current recommendations and the clinicians’ judgment. Treatment continuation was decided according to the clinical status and the evaluation of the imaging findings. To receive nivolumab, all patients had to have an Eastern Cooperative Oncology Group performance status < 2.

### Imaging protocol

Baseline PET imaging was performed within 1 month before the start of immunotherapy. Follow-up PET imaging was performed in all patients at approximately 8 weeks (equivalent to 4 cycles of treatment) after the first dose of nivolumab (SCAN-2). When warranted, a second follow-up FDG-PET was performed 4 weeks later (equivalent to 2 cycles of treatment) to confirm disease progression (SCAN-3).

PET/CT images were acquired 60 min after intravenous injection of FDG at a dose of 3 MBq/kg. The serum glucose level was < 1.4 g/L at the time of injection in all patients. All PET images were acquired with a Gemini TF PET/CT scanner (Philips Medical Systems, the Netherlands). The Gemini TF is a time-of-flight-capable, fully 3D PET scanner with a 16-slice Brilliance CT scanner. CT images were obtained without injection of contrast medium and with the following settings: 120 kV, 100 mA, 16 × 1.5 mm collimation, 0.69 pitch, 3 mm slice thickness, and 1.5 mm increments. PET images were reconstructed by using a BLOB-OS-TF list-mode iterative algorithm with 2 iterations and 33 subsets. A single-scatter simulation model was used for scatter correction. Attenuation correction was based on the CT data. No post-reconstruction smoothing filter was used. The reconstructed spatial resolution was 5 mm in the center of the field of view. The image voxel size was 4 × 4 × 4 mm for PET images and 1.17 × 1.17 × 1.5 mm for CT images. Peak standardized uptake values normalized by lean body mass (SULpeak) was calculated in a 1-cm-diameter region of interest placed on the highest uptake site of the tumor. Mean liver SUL was measured in a 3-cm-diameter spherical ROI as recommended in PERCIST [13].

### Metabolic response assessment

Images were centrally and jointly reviewed by two experienced nuclear physicians who had no knowledge of the patients’ medical history or symptoms (LG and MS). We introduced the concept of an iPERCIST classification, adapted from PERCIST and iRECIST [8, 13]. The objective was to obtain a reliable tool for assessing the response to immunotherapy, taking into account the possible avid-FDG lymphocytic tumoral infiltration under treatment. As recommended in PERCIST, baseline and follow-up scans were reviewed, and the lesion with the highest FDG uptake on each of these two scans was identified [14]. Complete metabolic response (CMR), partial metabolic response (PMR), and stable metabolic disease (SMD) were defined according to PERCIST [14]. CMR was defined as the visual absence of pathological FDG uptake in all baseline lesions identified on the baseline PET scan. PMR was defined as a decrease of at least 30% in the SULpeak of the target lesion, which is associated with an absence of progression of the non-target lesions. SMD was defined as an absence of response or progression.

Progressive metabolic disease (PMD) was defined as an increase of at least 30% in the SULpeak of the target lesion or as an unequivocal progression of the non-target lesions, regardless of the response in the target lesion. However, regarding PMD, we accounted for unconfirmed and confirmed progressive disease, from iRECIST [8], and established their corollaries with metabolic assessments. We defined two new categories replacing the PMD category: unconfirmed progressive metabolic disease (UPMD) and confirmed progressive metabolic disease (CPMD). UPMD was a PMD at SCAN-2, and CPMD was an UPMD confirmed 4 weeks later at SCAN-3. In iPERCIST, SCAN-3 is compared to SCAN-2, and patients were classified as CMR, PMR, SMD, or CPMD according to PERCIST recommendations. The continuation of treatment after the first evaluation by SCAN-2 was according to the physician’s judgment, which integrated metabolic response and the deterioration of clinical status, as described in iRECIST [8].

Finally, we classified patients as responders or non-responders. Patients with CMR, PMR, SMD, or UPMD followed by PMR or SMD were classified as responders. Patients with UMPD associated with clinical deterioration or UPMD followed by CPMD were classified as non-responders. These criteria are presented in Table 1.

**Table 1:** Comparison of RECIST 1.1, iRECIST, PERCIST, and iPERCIST criteria for immunotherapy evaluation

	RECIST 1.1	iRECIST	PERCIST	iPERCIST
Complete response	Disappearance of all target and non-target lesions nodes, must regress to < 10 mm in the short axis		Complete resolution of FDG uptake within the target lesion	
Partial response	≥ 30% decrease in tumor burden compared to baseline (largest diameter in axial plane)		≥ 30% decrease in the target tumor FDG SULpeak	
Stable disease	Neither partial response, complete response nor progressive disease		Neither partial response, complete response nor progressive disease	
Disease progression	≥ 20% + 5 mm absolute increase in tumor burden compared with nadir. Appearance of new lesions or progression of non-target lesions	≥ 20% + 5 mm absolute increase in tumor burden compared with nadir. Appearance of new lesions or progression of non-target lesions. (iUPD) Need to be confirmed 4–8 weeks later (iCPD); if progression is followed by tumor shrinkage, the bar is reset. Clinical stability is considered when deciding whether treatment is continued after iUPD	≥ 30% increase in FDG SULpeak or advent of new 18F-FDG-avid lesions	≥ 30% increase in FDG SULpeak or advent of new 18F-FDG-avid lesions (UPMD) Need to be confirmed by a second PET at 4–8 weeks later (CPMD); if progression is followed by PMR or SMD, the bar is reset. Clinical stability is considered when deciding whether treatment is continued after UPMD

*RECIST* Response Evaluation Criteria in Solid Tumors, *iRECIST* Immune RECIST, *PERCIST* PET Response Criteria in Solid Tumors, *iPERCIST* Immune PERCIST, *FDG* fluorodeoxyglucose, *iCPD* immune confirmed progressive disease, *iUPD* immune unconfirmed progressive disease, *UPMD* unconfirmed progressive metabolic disease, *CPMD* confirmed progressive metabolic disease, *PMR* partial metabolic response, *SMD* stable metabolic disease

In addition, we classified patients according to iRECIST criteria based on the CT (without contrast) part of the PET/CT scan [8] (see Table 1).

### Statistical analysis

Overall survival (OS) was defined as the time from the start of immunotherapy to death from any cause. The 1-year survival rate in responders and non-responders was recorded. We compared OS and the 1-year survival rate between responders and non-responders. The Kaplan-Meier method was used to estimate survival probability, with the level of significance estimated by the log-rank test. The difference between the continuous data for the two groups was determined by the Mann-Whitney test. Statistical analyses were performed using R Core Team (2014) software. The results were considered statistically significant when *p* <  0.05.

## Results

### Patient characteristics

**Fig. 1:**
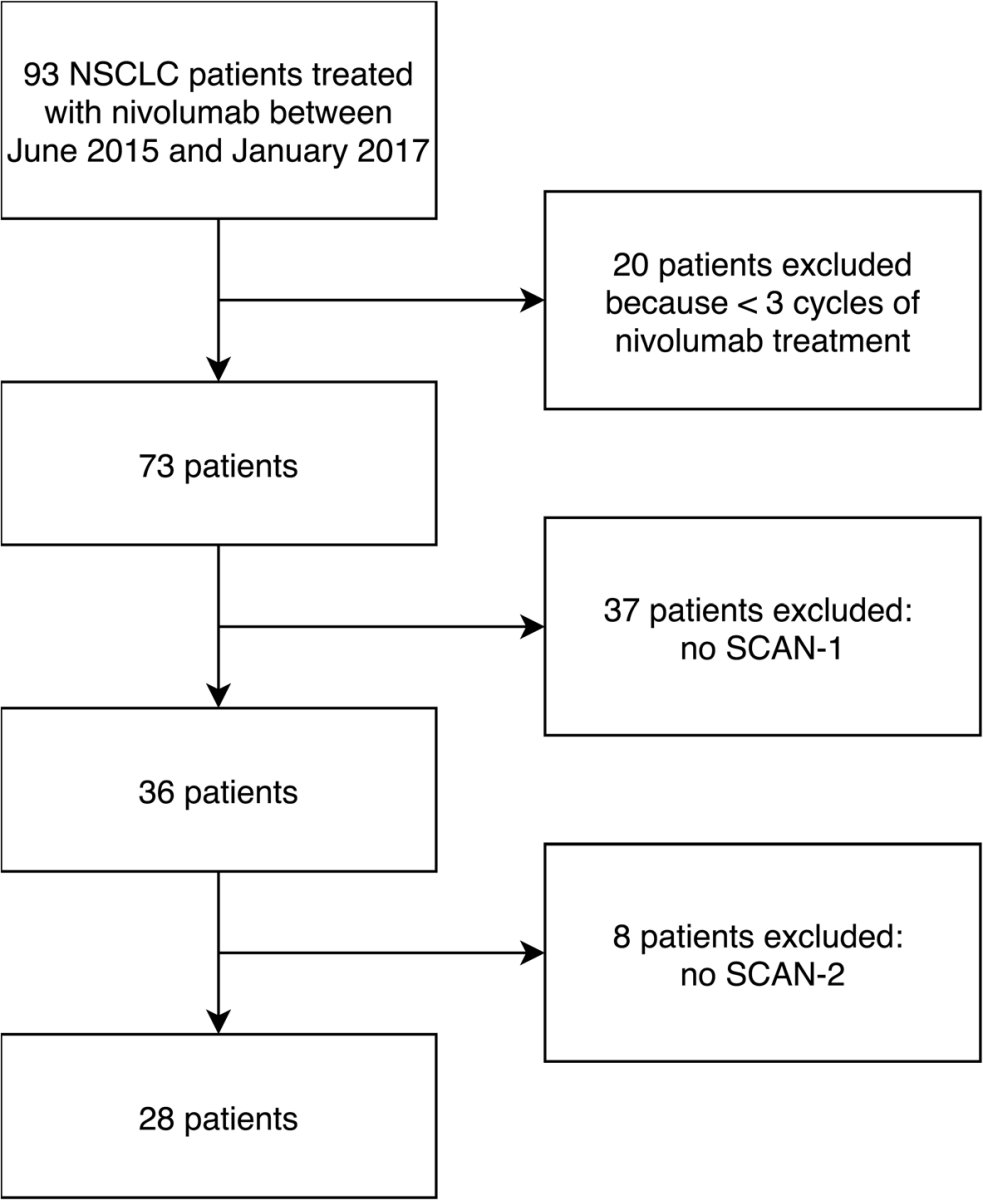
Flow chart of the screened population

A total of 28 patients (18 men; median age 63 years [range 42–79 years]) were retrospectively included (Fig. 1). Patient demographics are shown in Table 2. Briefly, 23 patients (82%) had a smoking history, adenocarcinomas were the most common histological tumor type, 23 tumors (82%) were initially metastatic, 5 patients (18%) were treated with surgery before disease recurrence, and patients received a median of 12 cycles of nivolumab.

**Table 2:** Patient characteristics (*n* = 28)

Characteristics	Nb (%)
Sex	
Male (%)	18 (64)
Female (%)	10 (36)
Age (median, range), years	63 (42–79)
Tobacco use (%)	23 (82)
Histology^a^	
Adenocarcinoma (%)	21 (75)
Squamous cell carcinoma (%)	4 (14)
Large cell carcinoma (%)	1 (4)
Large cell neuroendocrine carcinoma (%)	1 (4)
Lymphoepithelioma-like carcinoma (%)	1 (4)
Previous lines of chemotherapy (median, range)	2 (1–5)
Previous thoracic surgery	5 (18)
No. of nivolumab cycles (median, range)	11 (4–27)

^a^According to the 2015 World Health Organization Classification of Lung Tumors [26]

### Anti-PD-1 therapy evaluation with iPERCIST

**Fig. 2:**
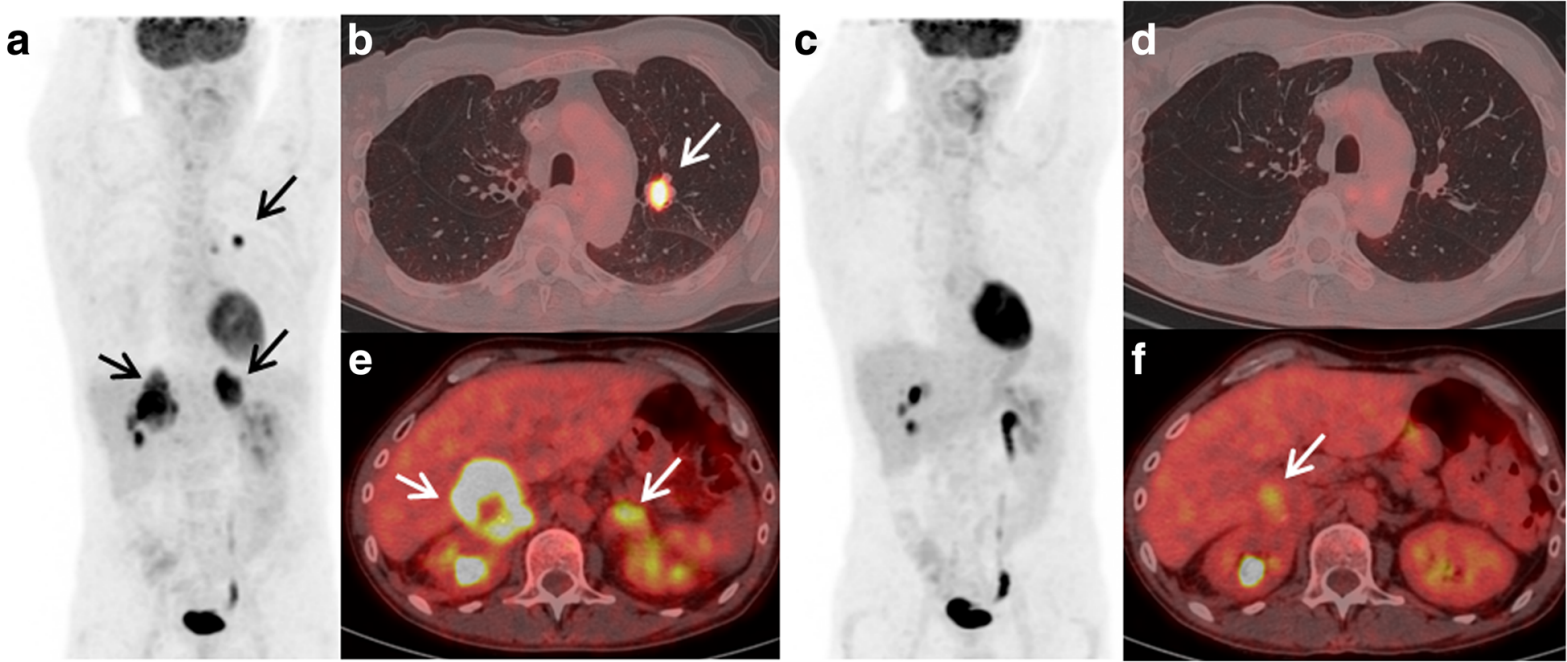
A 64-year-old patient with metastatic lung adenocarcinoma was treated with a second line of nivolumab. SCAN-1 shows lung nodules with adrenal metastases (**a**, maximum intensity projection (MIP) image, arrows; **b**, **e** axial fusion). SCAN-2 at 8 weeks of treatment (4 cycles) showing partial metabolic response (PMR): complete metabolic response on the lungs and left adrenal lesions (**c** MIP, **d** axial fusion) but persistent and significant FDG uptake on the right adrenal lesion (**f** arrow). The classification was PMR according to iPERCIST. Nivolumab treatment was maintained for 10 months, and at the last follow-up, the patient was still alive (survival of 23.8 months)

**Fig. 3:**
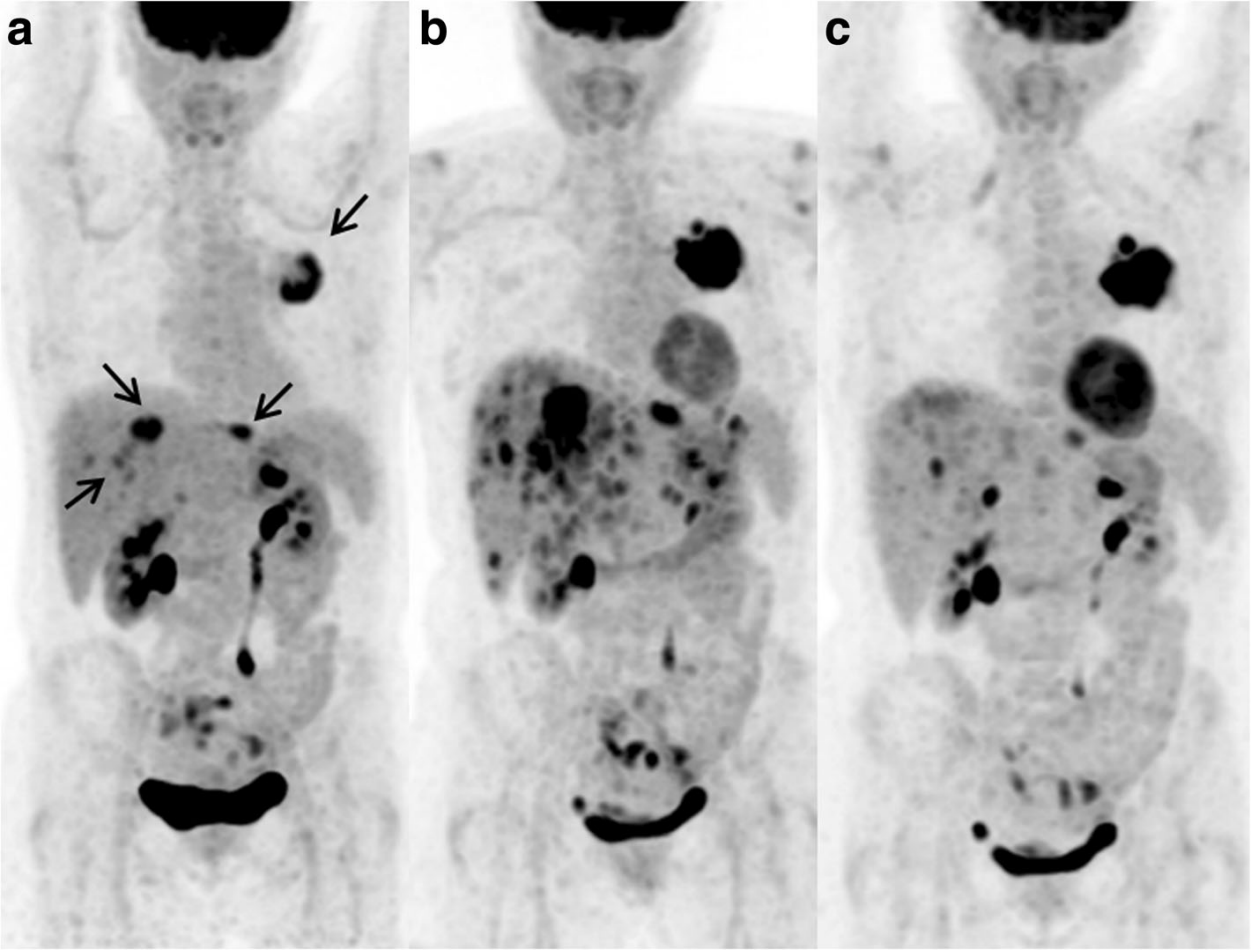
A 45-year-old women with metastatic lymphoepithelioma-like lung carcinoma treated with a second line of nivolumab. SCAN-1 showed left lung carcinoma with liver metastases (**a** maximum intensity projection image, MIP, arrows). SCAN-2 at 8 weeks of treatment (4 cycles) showed UPMD: increase in size of the primary lesion and appearance of numerous liver lesions (**b** MIP). SCAN-3 after two more cycles of nivolumab showed the disappearance of most liver lesions and a persistent large lung mass (**c** MIP). See Additional file 1: Figure S1 for axial views. Pseudo-progression was retrospectively diagnosed, and the classification was PMR according to iPERCIST. Nivolumab treatment was maintained for 7.1 months, and survival was 13.8 months

SCAN-2 was performed after 4 cycles of nivolumab in 20/28 patients, 3 cycles in 4/28 patients, 5 cycles in 3/28 patients, and 6 cycles in 1/28 patient. For the 28 patients at SCAN-2, 9 showed PMR (Fig. 2), 4 showed SMD, 2 showed CMR, and 13 showed UPMD. For the 2 patients with CMR, nivolumab treatment was maintained for 15.3 and 18.6 months, and the patients were still alive at the last follow-up. Among UPMD patients (13/28), only 4 were classified with SMD (*n* = 3) or PMR (*n* = 1) at SCAN-3 (Fig. 3 and Additional file 1: Figure S1). The remaining 9/13 UPMD patients showed clinical deterioration at the time of PET evaluation; thus, clinicians waived SCAN-3. Of these nine patients, five received a new chemotherapy line (docetaxel, gemcitabine, or erlotinib), three were referred for palliative care, and one was lost at follow-up.

Regarding semi-quantitative analysis with iPERCIST, we monitored the SULpeak of the lesion with highest uptake at SCAN-1 and SCAN-2. The average decrease in SULpeak of the highest uptake lesion was 75% for patients with CMR, 53% for patients with PMR, and 5% for patients with SMD. Among non-responders, SULpeak increased by 55%, on average. In 4/28 patients, we were unable to monitor the SULpeak because the difference between pre-treatment and post-treatment hepatic and blood pool SUL was > 20% (PERCIST recommendation [14]). Among these four patients, two were classified as UPMD due to the appearance of numerous lesions, one was classified as PMR due to the disappearance of all lesions except one, and one was classified as SMD because of the stability of the SULpeak as well as in the size of the lesions.

**Fig. 4:**
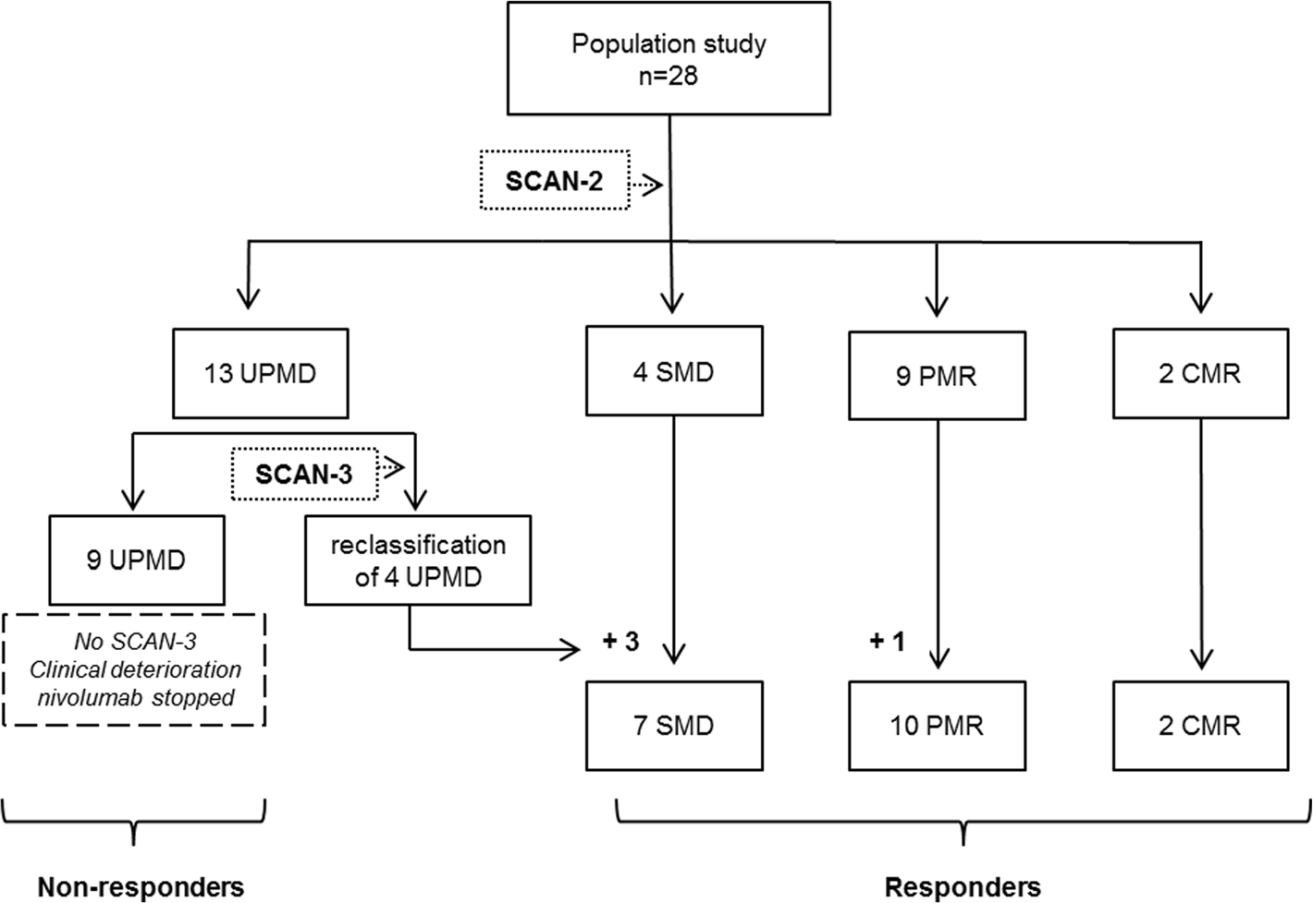
Category of imaging response according to iPERCIST

iPERCIST assessments in the population study are shown in Fig. 4 (responders *n* = 19, non-responders *n* = 9). Responders and non-responders did not differ in demographic characteristics (Table 3).

**Table 3:** Characteristics of patients responding and not responding to nivolumab treatment according to iPERCIST

	iPERCIST responders (*n* = 19)	iPERCIST non-responders (*n* = 9)	*p* value
Male (%)	13 (68)	4 (44)	0.6775
Tobacco use (%)	16 (84)	7 (78)	0.99
Age (mean, range), years	64 (42–79)	58 (50–79)	0.22
Adenocarcinoma (%)	14 (74)	7 (78)	0.99
Lines of chemotherapy before nivolumab (mean, range)	1.8 (1–5)	2.0 (1–5)	0.35
Survival (months, median)	19.9	3.6	< 0.0001

### Anti-PD-1 therapy evaluation with iRECIST

Evaluation with iRECIST was also conducted. For the 28 patients, evaluation at SCAN-2 showed PR in 5 patients, SD in 8 patients, CR in 2 patients, and UPD in 13 patients. Among the UPD patients (13/28), 4 were classified with SMD at SCAN-3.

Differences in iPERCIST classification were observed in 11/28 patients. Table 4 illustrates the modification in classification between the two criteria. In total, 5/11 patients were reclassified as PMR instead of as SD with iPERCIST, with a corresponding mean survival higher than the mean survival of the whole population (520 vs 479 days). Two patients were reclassified as PMD instead of as SD, two as SMD instead of as PD, one as CMR instead of as PR, and one as PMR instead of as CR.

**Table 4:** Differences between iRECIST and iPERCIST classification, observed in 11/28 patients, in relation to survival

iRECIST	iPERCIST	Nb of patients	Survival in days, mean (± SD)^a^
SD	PMR	5	520 (± 104)
SD	PMD	2	360 (± 234)
PD	SMD	2	573 (± 204)
PR	CMR	1	967
CR	PMR	1	717

*iRECIST* Immune RECIST, *iPERCIST* Immune PERCIST, *SD* Stable disease, *PD* progressive disease, *PR* partial response, *CR* complete response, *PMR* partial metabolic response, *PMD* progressive metabolic disease, *SMD* stable metabolic disease, *CMR* complete metabolic response

^a^NB: mean survival for the population study (*n* = 28) = 479 days (± 248)

### Survival analysis

**Fig. 5:**
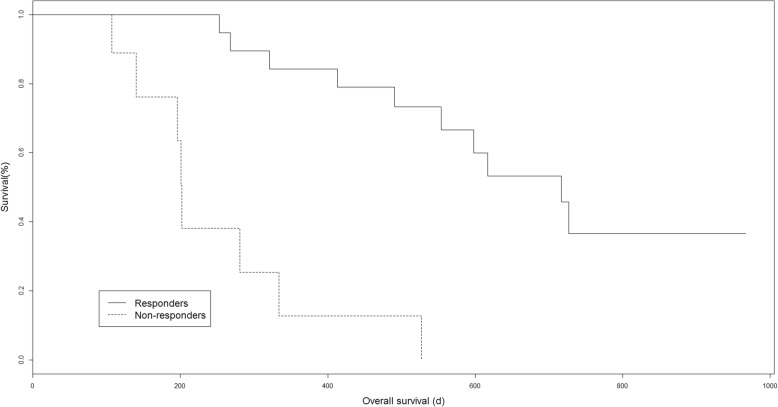
Kaplan-Meier curves for overall survival for metabolic responders vs non-responders (*p* = 0.0003)

The median follow-up was 16.7 months (range 3.6–32.2). At the last follow-up, 2/28 patients were still receiving nivolumab. For the other 26 patients, nivolumab was administered for a median of 6.6 months (range 1.1–26.3). Overall, 18/28 patients died during follow-up, and 10 patients were still alive at the end of follow-up (2 patients were still receiving nivolumab at the end of data collection). Responders continued treatment for a mean of 10.7 months (range 3.8–26.3). The 1-year survival rate was 94% for responders and 11% for non-responders*.* Additionally, OS significantly differed between responders and non-responders (19.9 and 3.6 months, *p* = 0.0003; Fig. 5). OS curves with iRECIST are shown in Additional file 2: Figure S3.

### PET studies evaluating treatment toxicities

One patient had thyroiditis at SCAN-2 (1/28). After the third injection of nivolumab, the thyroid stimulating hormone level decreased from 1.36 to 0.18 mUI/L (normally > 0.27 mUI/L). The patient did not show any symptoms. We did not observe any other features suggestive of adverse autoimmune disease associated with nivolumab (colitis, pancreatitis, hypophysitis, etc.).

## Discussion

In this study, FDG PET monitoring with iPERCIST was an effective tool for discerning NSCLC patients who could benefit from treatment with nivolumab. For responders to nivolumab according to iPERCIST (CMR, PMR, or SMD at SCAN-2, or with pseudo-progression confirmed by SCAN-3), the 1-year survival rate was greater than 90%, against 11% for non-responders. Additionally, OS was better for responders than non-responders at 19.9 vs. 3.6 months, *p* = 0.0003. Therefore, the prognostic value of iPERCIST might help physicians monitor immunotherapy in NSCLC patients.

FDG PET is currently the most widely used molecular imaging modality in clinical practice for staging and restaging NSCLC. However, few data are available for evaluating immunotherapy with FDG PET, especially in lung cancer, for which anti-PD-1 or anti-PDL-1-based immunotherapies are taking a crucial role in treating locally advanced or metastatic tumors [15]. Moreover, because the antineoplastic activity of immunotherapy is related to the activation of T cells against tumor cells, FDG accumulation might cause false-positive findings, as was underlined in RECIST 1.1, evolving the criteria toward iRECIST [8]. Consequently, implementing and evaluating PET-based criteria for immunomodulatory therapy [16] is needed.

We proposed to use iPERCIST derived from PERCIST, which was introduced by R. Wahl in 2009 [13, 14]. We modified iPERCIST by introducing two new categories of response derived from iRECIST: UPMD and CPMD, indicating that all metabolic progression observed at 8 weeks should be confirmed by another PET study 4 weeks later. However, the decision to continue immunotherapy treatment after the first evaluation is based on both clinical and imaging data. As recommended and discussed in the paper from Seymour et al. [8], the continuation of treatment beyond imaging progression (UPMD in iPERCIST) is permitted in patients who are clinically stable until the next assessment. Pseudo-progression is a rare but clearly described condition under PD1 inhibitor treatment in lung cancer [17]. At the time of the study, most of the described cases of pseudo-progression in lung cancer occurred in patients with clinical improvement or stabilization. The available data suggested that the UPMD patients with clinical deterioration had a progressive disease. Although these patients had no SCAN-3, they were followed after stopping immunotherapy; 6/9 UPMD patients showed tumoral progression on their CT scan approximately 2 months after the start of salvage chemotherapy, 2/9 patients died shortly (approximately 1 month) after the stopping nivolumab due to sepsis, and one patient had passed away at follow-up. Nevertheless, since the end of our study, a few case reports of patients with initial clinical worsening followed by a durable response were reported [18].

One important point that should be highlighted is that SMD patients after UPMD are considered metabolic responders with iPERCIST in our study. Indeed, we observed that most SMD patients evaluated by PET after 4–6 cycles of nivolumab treatment had a possible sustained metabolic response. As illustrated in the survival curve in Additional file 3: Figure S2, OS did not significantly differ between SMD patients and CMR + PMR patients when evaluated by iPERCIST.

The comparison of the results obtained with iRECIST and iPERCIST suggests that iPERCIST might be more relevant than iRECIST in terms of prognostic information. Indeed, 11/28 patients (39%) were classified differently with iPERCIST. In a review of the 2016 literature (6 series, 268 patients), discrepancies between RECIST and PERCIST were observed in approximately 38% of tumor responses, in line with our rate of disagreement [19]. In our study, PERCIST allowed five morphologically stable patients to be reclassified as PMR. The OS of these five patients was longer than the OS of the whole study population (520 vs. 459 days, respectively). Similarly, 2/11 patients were reclassified from progressive disease to stable disease, with their OS longer than the OS of the whole study population (573 vs. 459 days, respectively). Two other morphologically stable patients were reclassified as PMD, with their OS shorter than the OS of the whole study population (360 vs. 459 days). These data suggest that reclassification using PERCIST provides additional prognostic information compared to iRECIST in this setting.

Several studies have documented the value of FDG PET in patients receiving nivolumab, whether with large-cell B lymphomas [20] or melanomas [9]. In NSCLC, Kaira et al. recently investigated the role of FDG PET as a biomarker of therapeutic efficacy in a prospective study of 24 patients [21]. The authors showed that changes in FDG uptake evaluated by total lesion glycolysis 1 month after the start of nivolumab had significant prognostic value. Of note, SUVmax or metabolic tumor volumes failed to have prognostic value in multivariate analyses in their study. In addition, PERCIST using SULpeak was not evaluated in their study, although we considered PERCIST to be the most reliable criteria available. Indeed, the superiority of PERCIST over SUVmax has been demonstrated, especially in NSCLC treated with radiotherapy [22] or chemotherapy [23].

However, other authors showed that increased FDG uptake during immunotherapy predicted immune activation and could predict the best overall response [9]. Cho et al. studied the prognostic value of FDG PET 1 month after the start of treatment in patients with melanoma. In patients with stable disease at month 1 according to RECIST 1.1, PET could differentiate between two populations where an increase of 15.5% in SULpeak of the lesion with the highest uptake was associated with eventual clinical benefit (i.e., partial or complete response at 4 months or stable disease ≥ 6 months). The authors interpreted their findings by suggesting that an early inflammatory response at the site of the tumor, induced by boosting immunity, is a marker of a sustained response. This study lacked additional PET imaging after SCAN-2 to confirm the presence of an immune reaction rather than tumoral progression, which could explain the low accuracy and negative predictive value of PERCIST (70% and 42%, respectively) for predicting the best overall response after 4 months, because of the possible misclassification of pseudo-progression in PMD.

Recent studies of melanoma patients receiving ipilimumab and those evaluated by FDG PET suggested the use of new criteria for imaging evaluation [10, 11]. The PET Response Evaluation Criteria for Immunotherapy (PERCIMT) added new criteria defining a threshold of two to four new lesions in an evaluation scan to classify patients under immunotherapy as PMD. Again, a single-time-point evaluation here does not seem reliable enough in the setting of immunotherapy and can lead to a risk of misclassifying pseudo-progression as disease progression. This issue was observed in initial observations, which found that 10% of patients who received ipilimumab for melanoma showed a clinical response (including partial response and stable disease) that would have been misclassified as disease progression by World Health Organization criteria [24]. Using FDG PET, we assumed that the risk of misclassification might be even higher because of the better sensitivity of FDG PET, particularly for detecting small lymph nodes or bone metastasis. Notably, we identified 1 patient (1/28, 4%) with a pseudo-progression, which is in accordance with the rate from the literature that ranges from 0 to 6% [25].

Our study is mainly limited by its retrospective design and potential recruitment bias. Of note, the response rate in our study was higher than that usually reported with studies of nivolumab at 40% compared with 20% reported in a recent publication [4]. The choice of the most relevant imaging test evaluation in the context of immunotherapy treatment is an important issue, particularly considering that CT scans are less expensive and more widely available compared to PET scans. Prospective studies will need to validate the cost-effectiveness of a whole-body metabolic evaluation with FDG PET compared to conventional anatomical assessment. Finally, we did not compare FDG PET with predictive biomarkers, such as PD-L1 expression in tissue sampling [16], because of its unavailability at the time of the study.

## Conclusions

FDG PET might be a powerful tool for evaluating the response to anti-PD-1-based immunotherapy, with the ability to identify patients who will benefit the most from treatment. The prognostic value of the newly introduced iPERCIST should be confirmed in large prospective multicentric studies.

## Additional files

Additional file 1: **Figure S1.** 45-year-old women with metastatic lymphoepithelioma-like lung carcinoma treated with a second line of nivolumab. (DOCX 440 kb)

Additional file 2: **Figure S3.** Kaplan-Meier curves of overall survival for iRECIST groups: CR+PR, SD and PD (log rank test, *p*=0.023). (DOCX 67 kb)

Additional file 3: **Figure S2.** Kaplan-Meier curves for overall survival with stable metabolic disease (SMD) vs. partial metabolic response (PMR) + complete metabolic response (CMR) (p=0.78). (DOCX 63 kb)

## Abbreviations

CMR: Complete metabolic response
CPMD: Confirmed progressive metabolic disease
CT: Computed tomography
FDG: Fluorodeoxyglucose
iPERCIST: Immune PET Response Criteria in Solid Tumors
iRECIST: Immune Response Evaluation Criteria in Solid Tumors
NSCLC: Non-small cell lung carcinoma
PET: Positron emission tomography/computed tomography
PMD: Progressive metabolic disease
PMR: Partial metabolic response
RECIST: Response Evaluation Criteria in Solid Tumors
SMD: Stable metabolic disease
SULpeak: Peak standardized uptake values normalized by lean body mass
UPMD: Unconfirmed progressive metabolic disease
