# Supplementary material for: Monitoring anti-PD-1-based immunotherapy in non-small cell lung cancer with FDG PET: introduction of iPERCIST
Source: EJNMMI Res. 2019 Jan 29;9:8. doi: 10.1186/s13550-019-0473-1 (PMC6890907; doi:10.1186/s13550-019-0473-1)

**Figure S1**

45-year-old women with metastatic lymphoepithelioma-like lung carcinoma treated with a second line of nivolumab. SCAN-1 showed left lung carcinoma with liver metastases (a and d, axial fusion). SCAN-2 at 8 weeks of treatment (4 cycles) showed UPMD: increase in size of the primary lesion (b) and appearance of numerous liver lesions (e). SCAN-3 after 2 more cycles of nivolumab showed disappearance of most liver lesions (f) and a persistent large lung mass (c). Pseudo-progression was retrospectively diagnosed and the classification was PMR according to iPERCIST. Nivolumab treatment was maintained for 7.1 months and survival was 13.8 months.


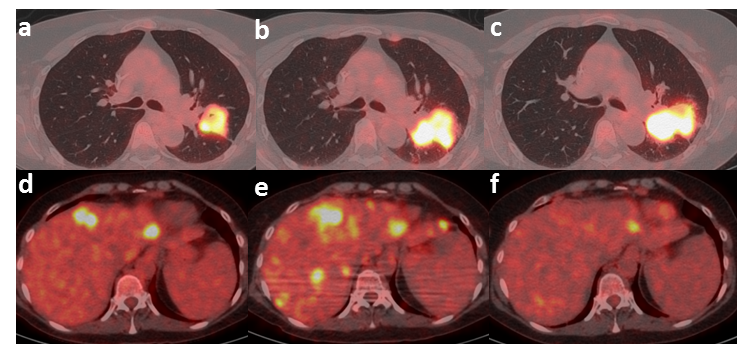

Supplement: Supplementary file 1 — Figure S1. 45-year-old women with metastatic lymphoepithelioma-like lung carcinoma treated with a second line of nivolumab. (DOCX 440 kb) [file 13550_2019_473_MOESM1_ESM.docx]
