# Supplementary material for: Monitoring anti-PD-1-based immunotherapy in non-small cell lung cancer with FDG PET: introduction of iPERCIST
Source: EJNMMI Res. 2019 Jan 29;9:8. doi: 10.1186/s13550-019-0473-1 (PMC6890907; doi:10.1186/s13550-019-0473-1)

**Figure S3.** Kaplan-Meier curves of overall survival for iRECIST groups: CR+PR, SD and PD (log rank test, p=0.023).


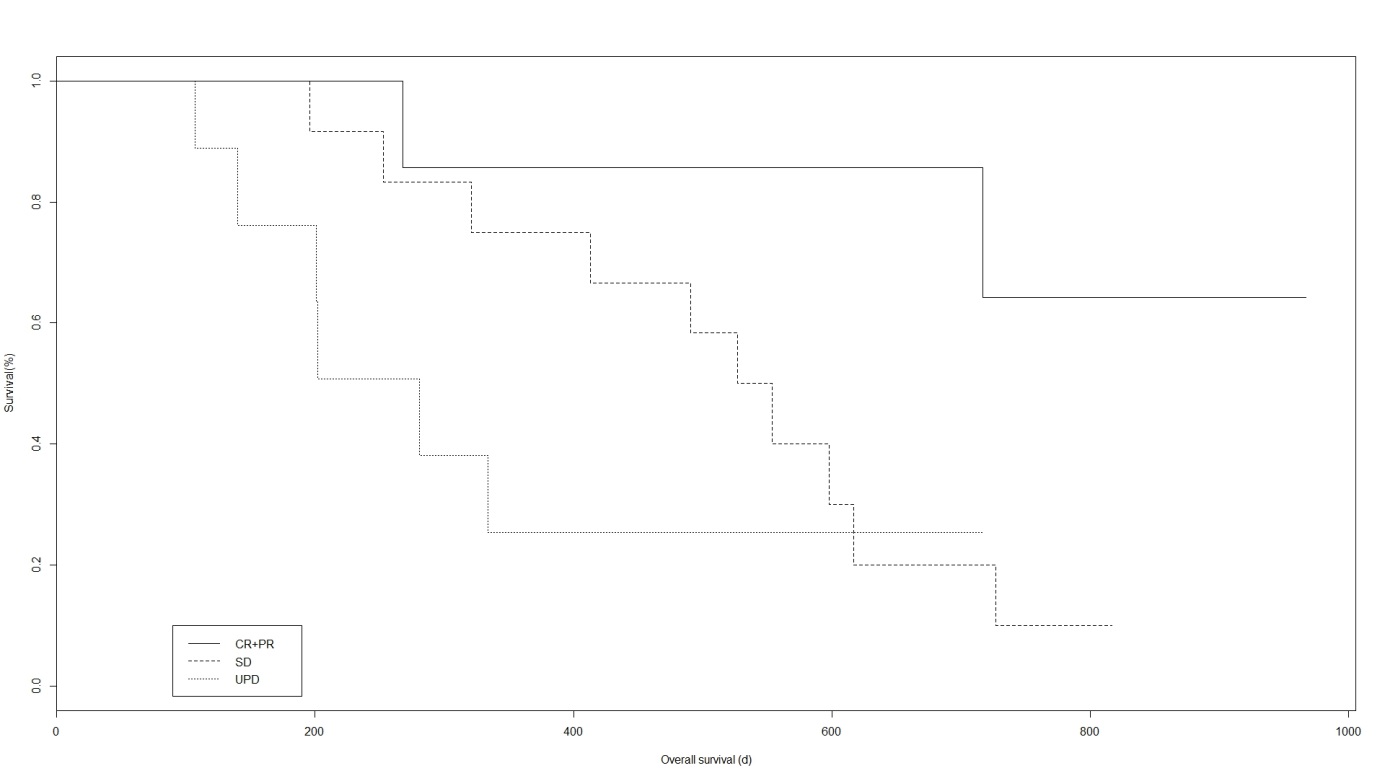

Supplement: Supplementary file 2 — Figure S3. Kaplan-Meier curves of overall survival for iRECIST groups: CR+PR, SD and PD (log rank test, p=0.023). (DOCX 67 kb) [file 13550_2019_473_MOESM2_ESM.docx]
