# Supplementary material for: Monitoring anti-PD-1-based immunotherapy in non-small cell lung cancer with FDG PET: introduction of iPERCIST
Source: EJNMMI Res. 2019 Jan 29;9:8. doi: 10.1186/s13550-019-0473-1 (PMC6890907; doi:10.1186/s13550-019-0473-1)

**Figure S2**

Kaplan-Meier curves for overall survival with stable metabolic disease (SMD) vs. partial metabolic response (PMR) + complete metabolic response (CMR) (p=0.78).


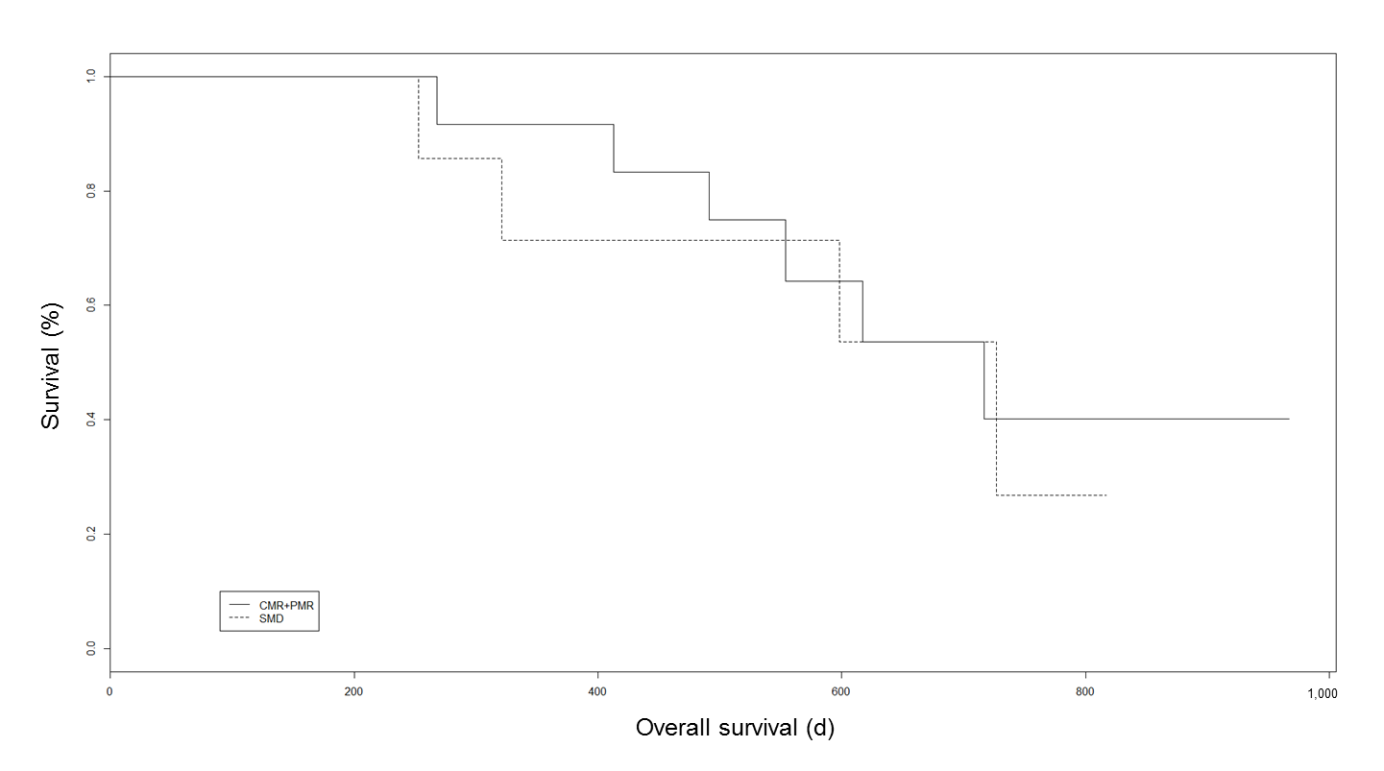

Supplement: Supplementary file 3 — Figure S2. Kaplan-Meier curves for overall survival with stable metabolic disease (SMD) vs. partial metabolic response (PMR) + complete metabolic response (CMR) (p=0.78). (DOCX 63 kb) [file 13550_2019_473_MOESM3_ESM.docx]
